# Cervicocephalic kinesthetic sensibility and postural balance in patients with nontraumatic chronic neck pain – a pilot study

**DOI:** 10.1186/1746-1340-17-6

**Published:** 2009-06-30

**Authors:** Per J Palmgren, Daniel Andreasson, Magnus Eriksson, Andreas Hägglund

**Affiliations:** 1 Department of Research, Scandinavian College of Chiropractic, Råsundavägen 101,169 57 Solna, Sweden; 2 Spinal Balans, Hälsingegatan 5, 113 23 Stockholm, Sweden; 3 Department of Preclinical studies, Scandinavian College of Chiropractic, Råsundavägen 101,169 57 Solna, Sweden

## Abstract

**Background:**

Although cervical pain is widespread, most victims are only mildly and occasionally affected. A minority, however, suffer chronic pain and/or functional impairments. Although there is abundant literature regarding nontraumatic neck pain, little focuses on diagnostic criteria. During the last decade, research on neck pain has been designed to evaluate underlying pathophysiological mechanisms, without noteworthy success. Independent researchers have investigated postural balance and cervicocephalic kinesthetic sensibility among patients with chronic neck pain, and have (in most cases) concluded the source of the problem is a reduced ability in the neck's proprioceptive system. Here, we investigated cervicocephalic kinesthetic sensibility and postural balance among patients with nontraumatic chronic neck pain.

**Methods:**

Ours was a two-group, observational pilot study of patients with complaints of continuous neck pain during the 3 months prior to recruitment. Thirteen patients with chronic neck pain of nontraumatic origin were recruited from an institutional outpatient clinic. Sixteen healthy persons were recruited as a control group. Cervicocephalic kinesthetic sensibility was assessed by exploring head repositioning accuracy and postural balance was measured with computerized static posturography.

**Results:**

Parameters of cervicocephalic kinesthetic sensibility were not reduced. However, in one of six test movements (flexion), global repositioning errors were significantly larger in the experimental group than in the control group (*p *< .05). Measurements did not demonstrate any general impaired postural balance, and varied substantially among participants in both groups.

**Conclusion:**

In patients with nontraumatic chronic neck pain, we found statistically significant global repositioning errors in only one of six test movements. In this cohort, we found no evidence of impaired postural balance.

Head repositioning accuracy and computerized static posturography are imperfect measures of functional proprioceptive impairments. Validity of (and procedures for using) these instruments demand further investigation.

**Trial registration:**

Current Controlled Trials ISRCTN96873990

## Background

Cervical pain is common, affecting many people to varying degrees. It rarely is serious, and is often a consequence of several interacting factors with unknown etiology [1]. Neck pain can be acute, subacute, or chronic, pain or functional disability lasting for 0–4 weeks (acute), 4–12 weeks (subacute), or more than 12 weeks (chronic). Curing neck pain is challenging, but several therapies can help [1]. Chiropractic care and manipulative therapy has been shown to reduce soreness and improve function in patients with chronic neck pain of nontraumatic origin [2-4].

Over the last decade, functional impairment of suboccipital and deep cervical flexor muscles, and cervical mechanoreceptive dysfunction, have been thought to affect proprioception in necks of patients with chronic cervical pain [5]. Ability to reposition the head to a previous position is dependent on cervicocephalic kinesthetic sensibility [6], and a method for evaluating it was introduced by Revel et al. [7]. Movement of the head relative to the trunk involves information from the cervical proprioceptive apparatus and the vestibular system, the former perhaps playing a primary role [8]. Pinsault et al. [9] recently suggested that the vestibular system is probably not involved in returning the head to a neutral position in the cervicocephalic relocation test, and supported this test as a measure of cervical proprioceptive acuity. Disturbed kinesthetic sensitivity has been implicated in functional instability of joints, and their susceptibility to re-injury, chronic pain, and even degenerative disease [10]. Evidence also suggests that removal of deleterious or abnormal afferent input at the site of articulation alone may result in improved proprioception and motor response [11]. Some studies [3,7,12-17], although not all [5,18,19], have reported that impaired position sense, quantified by reduced head relocation accuracy and increased cervical joint position errors, is present in patients with traumatic and idiopathic (nontraumatic) neck pain.

While neck pain may alter proprioceptive function, there is no clear consensus in the literature. Furthermore, no general agreement has been reached on how to perform head repositioning tests, or dichotomize results. In a recent study of intra- and inter-examiner reliability, Strimpakos et al. [20] concluded that researchers measuring neck proprioception have failed to provide reliable measures and conclusive observations.

Chronic neck pain may be linked to reduced cervicocephalic kinesthetic sensibility and postural balance [21,22]. From a manual therapeutic viewpoint, this is appealing, as many manual diagnostic and therapeutic procedures detect these phenomena.

Among participants with chronic neck pain, investigators have used different static and dynamic measurements of balance to show significant abnormalities in standing vertical posture [16,21-23]. Persons suffering chronic neck pain tended toward joint dysfunction, muscle atrophy, and standing imbalance [24]. Reduced balance and amplified sway have also been reported in studies of patients with chronic neck pain with severe etiology, such as trauma or whiplash-associated disorders [23,25-27]. A number of mechanisms involved in neck pain might cause distorted cervical somatosensory input to the postural control system. Field et al. enumerated these as *direct trauma, inflammatory mediators*, and *effects of pain on nociceptors and mechanoreceptors *[16].

However, few studies have investigated sensorimotor control in nontraumatic neck pain, using head repositioning accuracy (as described by Revel et al. [7]), and vertical standing balance. This reflects a gap in understanding of cervical pain. Therefore, we aimed to investigate cervicocephalic kinesthetic sensibility and postural balance among patients with nontraumatic chronic neck pain. We hypothesized that they would show disturbed cervicocephalic kinesthetic sensibility (as measured by HRA), and altered postural control (measured using computerized static posturography).

## Methods

### Patient Selection

The study was performed at the Scandinavian College of Chiropractic in Stockholm, Sweden, using a two-group, observational design, with repeated measures. Participants were given oral and written information before agreeing to participate. The project was approved by the Research Ethical Board of the Chiropractic Association of Sweden, and the Scandinavian College of Chiropractic Scientific Council (board of ethical approval), in accordance with the Declaration of Helsinki.

Patients complaining of 3 months of ongoing neck pain (13 women and 2 men, mean age = 38.8 years; SD = 7.4) were recruited by convenience sampling, from the institutional outpatient clinic. Inclusion and exclusion criteria are listed in Table 1. Two persons were excluded due to earlier trauma and a misunderstanding of age criteria, leaving 13 participants. Sixteen healthy persons (6 women and 10 men, mean age = 35.1 years; SD = 5.0) were recruited to a control group.

**Table 1:** Criteria for patient inclusion and exclusion

**Inclusion**	1. Age 30–55 years
	2. Neck pain prolonged more than 12 weeks^1^
	
**Exclusion**	1. Neck trauma
	2. Received manual treatment within one week prior to the investigation
	3. Chronic low back pain (> 3 months)
	4. Arthrodesis in foot or ankle
	5. Evidence of impaired function/pain in foot or ankle
	6. Evidence of impaired function/pain in knee
	7. Evidence of impaired function/pain in hip
	8. Diastolic pressure > 110 mm Hg
	9. Pregnancy
	10. Drug abuse
	11. Aid for walking or standing
	12. Known disease that affects nervous system (e.g., multiple sclerosis, stroke, Parkinson disease)
	13. Known disease that affects vestibular apparatus (e.g., Meniére disease, benign paroxysmal positional vertigo)

^1 ^Not valid for participants in the control group

### Outcome Measures: head repositioning accuracy

Head repositioning accuracy (HRA) measures the ability of the neuromusculoskeletal system to reposition the head to a neutral posture, after movements in different planes. A cervical joint positioning error is considered to mainly reflect disturbed afferent input from articulations of the neck, and muscle receptors [11]. The test assesses the ability to perceive both movement and position of the head, relative to the trunk. Joint positioning error results in an angular difference between the starting position and the resumed neutral head posture. This angle can be measured as the distance between the starting point and the final position of a laser spot on a target sheet, projected from a subject's head. The dependent variable that reflects accuracy during head repositioning is most commonly measured in angular units (degrees) or linear metric units [28].

**Figure 1:**
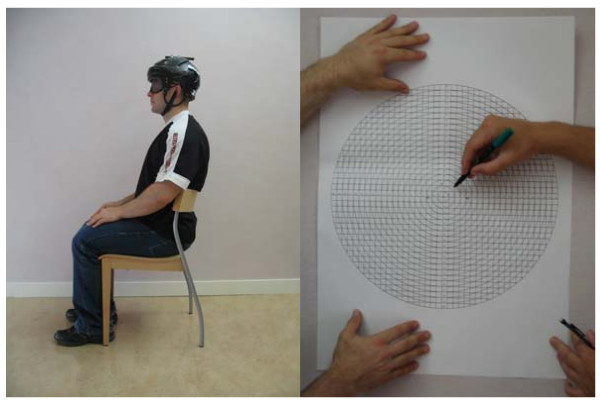
**Arrangement of participant for HRA procedure, helmet with pointer (left), and mobile coordinate system (right)**.

In our study, the same investigator measured HRA for all participants, and was blinded to group membership. Each participant was seated in a chair, with support for the lower back (Figure 1, left). Lateral aspects of the feet were placed 40 cm apart, using markings on the floor. Investigators also confirmed that each participant's thighs were horizontal, and knee joints flexed at 90°. A 648 g ice hockey helmet with an attached laser pointer was placed on the head of each participant, and adjusted for fit. The laser pointer was situated at a 90° angle to a 40 cm, mobile coordinate system (Figure 1, right), with numbered concentric circles for each centimeter along the radius. The HRA procedure and purpose were again explained to each participant. Eyes were occluded with a sleeping mask, and the participant was asked to keep eyes closed during the entire procedure. Following pre-recorded and standardized instructions, participants were asked to memorize their neutral head position, and to duplicate it after an active, slow phase, sub-maximal, specific movement of the head. Once the new "neutral" position was stabilized (time standardized by pre-recorded instructions), investigators registered the new location of the laser spot, and measured the distance to the starting position. With the participant now resting in a new neutral position, the mobile target was moved to reposition the laser spot at the center of the target, before a new head movement was requested. Each directional movement was repeated consecutively 10 times, and the average of assessments was used as a result in that direction. Head movements were done in left and right rotation in the horizontal plane, extension and flexion in the sagittal plane, and left and right lateral flexion in the frontal plane. Six head movements were performed, 10 times each, totaling 60 consecutively repeated movement-repositioning tasks designed to follow predictable paths of movement.

### Outcome Measures: computerized static posturography

Computerized static posturography (CSP) was used to assess balance. Under altered visual conditions, a stable force platform (model FP4, HUR labs Force Platform, Tampere, Finland, ) measured postural sway and changes in standing balance. The force platform measured 60 × 60 cm, with industrial grade force transducers at each corner. Ground reaction forces were registered, and changes over time were measured in both medial-lateral and anterior-posterior directions. Sensors had a measuring range of 0–200 kg. Force changes were sent by USB connection to a laptop computer (recorded using Windows 2000 [Microsoft, Seattle, WA]), and raw traces were produced both numerically and graphically. Accompanying software (Finsole Orthothic Analyzing Suite, HUR, Balance Software 1.23, Tampere, Finland) provided easy data acquisition and immediate analysis of results.

**Figure 2:**
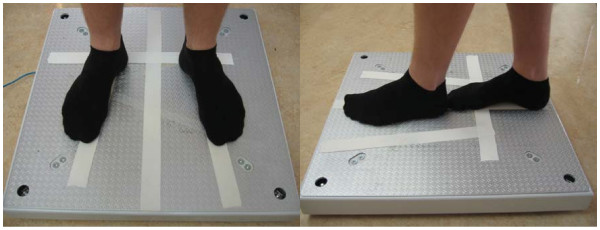
**Position of participant's feet in comfortable position (left) and tandem stance (right)**.

Postural performance was assessed in a calm, undisturbed room. Participants stood without shoes on the force platform, body in anatomical position and arms at their sides. Participants' feet were repositioned exactly on the force platform for every test, using platform foot markings and the investigator confirmed the foot positions prior, during and after each test session. Each participant was tested for static balance using a standing Romberg test for 60 seconds with eyes open, immediately followed by 60 seconds with eyes closed. In tests with eyes open, participants focused on a 5-cm diameter black dot, on a wall approximately 3 meters away, at the height of the participants' eyes. Participants were instructed to keep arms at their sides, and remain as still as possible during measurement. Tests performed (eyes open and closed) were: comfortable position (Figure 2, left) [27], and tandem stance (a more provocative test; Figure 2, right).

**Figure 3:**
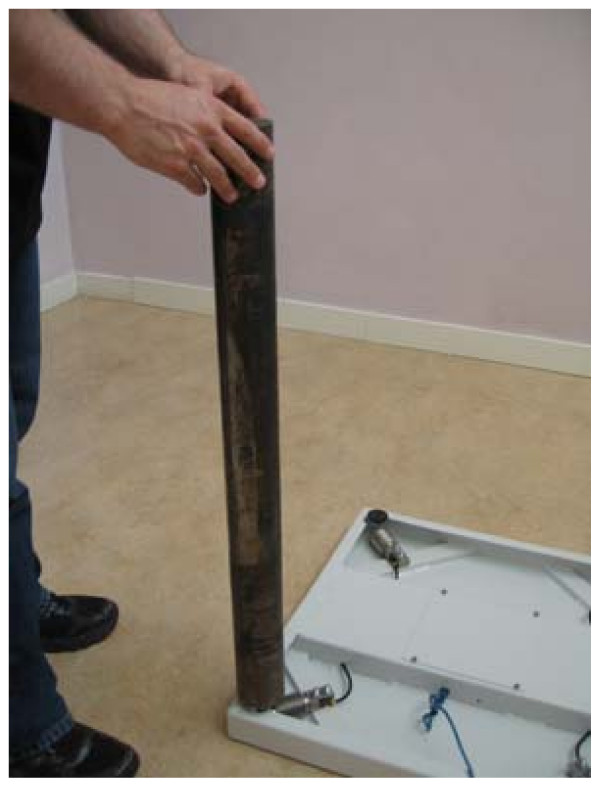
**Placement of weight during calibration of force platform**.

Prior to each trial, for all participants, the same investigator calibrated the force platform, according to manufacturer's recommendations and The Committee for Standardization of Stabilometric Methods and Presentations. This involved an 805 mm, 24.650 g, 70 mm diameter, metallic, calibration weight (Figure 3).

The CSP measured how the participant's center of pressure changed with time. Two values were collected for each registration: the total trace length/distance covered by the projection of the center of gravity (measured in millimeters), and 90% of the area enclosed by the track of the same projection (measured in millimeters squared).

To check for the possibility that individual measurements of postural balance would provide unreliable values, we examined procedural reliability by comparing values from one test sequence with means from three to five test sequences (with all conditions). When comparing groups, no significant differences could be detected that would indicate low procedural reliability that averaging of a greater number of repeat measurements would give more reliable results.

### Outcome Measures: both HRA and CSP

To help interpret sensorimotor function tests (HRA and CSP), a Visual Analogue Scale (VAS) was used to quantify participant pain at the time of investigation. All participants completed a VAS questionnaire regarding intensity of pain in their cervical region, by marking continuous, 100 mm, linear scales, with two extremes: no pain and worst imaginable pain. Test-retest reliability for the VAS has been reported (r = 0.99; *p *< .05) [29,30], and it may be a better psychometric instrument than the McGill Pain Questionnaire [31]. We collected no data on pre-investigation pain levels, such as pain during the preceding week, worst pain, or pain during specific tasks.

### Patient recruitment

All persons invited to participate agreed to do so, and inclusion and exclusion criteria were confirmed (Table 1). Exclusion criteria were designed to eliminate confounding ailments and injuries that might influence balance ability or the proprioceptive system in the neck.

To minimize participation selection bias, participants also underwent a brief, clinical investigation, consisting of a history and a clinical orthopedic screening: toe/heel walk for distal muscle function and movement; squat-test for proximal muscle function and movement; blood pressure; and vascular auscultation. This clinical investigation complemented exclusion criteria, clarified clinically functional status, and helped purge conditions that might influence outcome measures.

### Statistical analysis

The main variables compared between experimental and control groups were those deriving from HRA and CSP. Following testing for normal distribution (D'Agostino-Pearson normality test), socio-demographics and pain characteristics were compared using Fisher's exact test. For HRA, projections of the laser on the coordinate system (following movement) were measured (X, Y), and each coordinate was given a positive or negative value, according to its location in relation to the point of origin before repositioning. Using these two values, the participant's global HRA (radius) in centimeters was calculated trigonometrically. The mean value and standard deviation of the global error from zero for each component in the repositioning task was calculated for the 10 consecutive trials in each test movement, and used for data analysis. We used absolute value (magnitude only) in measuring deviation from the origin, rather than a positive or negative value; thus, no distinction was made between over- and underestimation of the original neutral position. The difference between the smallest measured distance from the origin to the final position after movement, and the largest measured distance from the origin were measured in both groups. Data from HRA and CSP were normally distributed, and differences were studied using an unpaired *t *test (2-tailed). Statistical analyses were performed using GraphPad Prism (version 5.00), and power calculations were done using GraphPad StatMate (version 2.00; GraphPad Software, San Diego, California, USA). Data analysis was performed by an independent statistician. Probability values less than 0.05 (5%) were considered statistically significant.

## Results

**Figure 4:**
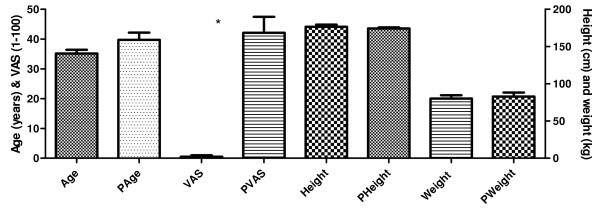
**Demographics (group mean ± SD for control and experimental (P) group, respectively) displaying age in years, height, VAS scores and weight**. VAS scores were statistically different between groups (*).

Distributions of age, weight, and height, and VAS scores are shown in figure 4. There were significant differences between groups in VAS, because no-one in the control group reported pain. There were no significant differences in age, height, or weight.

### Cervicocephalic kinesthetic sensibility (HRA)

**Figure 5:**
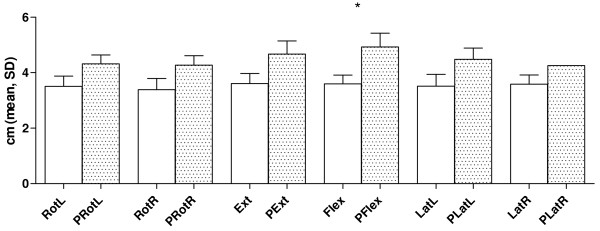
**Head Repositioning Accuracy (group mean ± SD for control and experimental (P) group, respectively) for different head movements**. (Ext = extension; Flex = flexion; LatL = left lateral flexion; LatR = right lateral flexion; PExt = patients extension; PFlex = patients flexion; PLatL = patients left lateral flexion; PLatR = patients right lateral flexion; PRotL = patients left rotation; PRot R = patients right rotation; Rot L = left rotation; Rot R = right rotation) measured for each individual as the mean of ten consecutively repeated movements in each direction. Only flexion was statistically different between groups (*).

Distributions within groups of reposition distances from the origin, following different movements, are displayed in Table 2. Differences between smallest and largest measured distances from the origin were large in both groups. The experimental group showed larger minimum distances in all aspects of HRA. In addition, maximum distances were larger for the experimental group in all movements except right lateral flexion. Flexion showed statistically significant differences between groups (*p *< .05), with a mean distance from the origin of 3.6 ± 1.3 cm in the control group and 5.1 ± 2.0 cm in the experimental group. No other significant differences could be detected between the groups. (Figure 5)

**Table 2:** Minimum and maximum values (cm) of the distance from the origin following different neck movements.

	**Rot L**		**Rot R**		**Ext**		**Flex**		**Lat L**		**Lat R**	
	
	min	max	min	max	min	max	min	max	min	max	min	max
Control group	1.5	6.3	1.4	6.0	1.8	6.4	1.4	6.2	1.5	6.3	1.6	6.4

Experimental group	3.2	6.9	2.6	7.3	2.5	7.9	2.1	8.9	3.1	7.8	3.1	6.3

(Ext = extension; Flex = flexion; Lat L = left lateral flexion; Lat R = right lateral flexion; Rot L = left rotation; Rot R = right rotation)

### Postural balance (CSP)

Lengths and ellipse areas associated with postural sway displayed extensive variations in all tests, both within and between groups.

Some results of tests with tandem stance and closed eyes were not obtainable, because participants stepped off the platform or lost their balance, which resulted in drop-outs from both groups. From the control group, data from 12 participants could be used, and from the experimental group, only seven.

**Figure 6:**
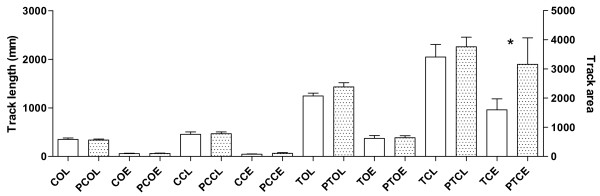
**Computerized Static Posturography (group mean ± SD for control and experimental group, respectively) for different static test positions for trace & ellipse lengths**. (CCE = comfortable position with closed eyes, elliptical area; CCL = comfortable position with closed eyes, trace length; COE = comfortable position with open eyes, elliptical area; COL comfortable position with open eyes, trace length; PCCE = patients comfortable position with closed eyes, elliptical area; PCOE = patients comfortable position with open eyes, elliptical area; PCCL = patients comfortable position with closed eyes, trace length; PCOL patients comfortable position with open eyes, trace length; PTCE = patients tandem stance with closed eyes, elliptical area; PTCL = patients tandem stance with closed eyes, trace length; PTOE = patients tandem stance with open eyes, elliptical area; PTOL = patients tandem stance with open eyes, trace length; TCE = tandem stance with closed eyes, elliptical area; TCL tandem stance with closed eyes, trace length; TOE tandem stance with open eyes, elliptical area; TOL = tandem stance with open eyes, trace length). Only TCE was significantly different between the groups (*).

For tandem stance with closed eyes, statistically significant differences between groups were detected in ellipse area (*p *< .05, *t *test). No other significant differences were detected. (Figure 6)

## Discussion

### Key findings

Compared to participants without neck pain, our limited sample did not indicate a general reduction of cervicocephalic kinesthetic sensibility among patients with chronic neck pain of nontraumatic origin. However, for flexion, global repositioning errors were significantly larger in the experimental group than in the control group (*p *< .05). For other movements, there were no significant differences in HRAs. Results from CSP measurements did not demonstrate any general impaired static posture among participants. Only one of eight parameters tested–ellipse area in tandem stance with closed eyes–showed significant differences between groups (*p *< .05). However, substantial variations were seen within and between groups.

### Methodological considerations

Lack of statistically significant differences in five of six HRA tests and nearly all CSP tests may be due to the small number of study participants. Following the study, analysis indicated that the power was only about 30% (range, 30% to 60%) for HRA and about 3% to 40% for CSP. Compared to a more desirable 80%, these values indicate that groups were not large enough to ensure that differences would be detected in our study, if present. Thus, we cannot be sure that so few differences exist between groups in HRA and CSP.

Another limitation was that we did not assess functional performance. Therefore, the experimental group may not have had a sufficient degree of pain and functional impairment to result in a detectable difference between groups.

### Comparison with Findings of Others

Although our sample was small, and HRA and CSP results varied among participants, one of six test movements for HRA showed significant differences, suggesting a possible interaction of some or several underlying mechanisms.

Although chronic neck pain can be defined in clinical terms, underlying pathophysiological mechanisms are still primarily unidentified. As with chronic low back pain, investigations have failed to demonstrate a consistent relation linking structural pathology and neck-related pain [32-37]. There are no clear criteria for how chronic neck pain should be diagnosed and classified [1]. Furthermore, large inherent variations in functional proprioceptive impairment and pain within one group of patients with nontraumatic neck pain might contribute to the variety of results.

Our findings are consistent with studies reporting no significant impairment of kinesthesia in patients with nontraumatic neck pain, or whiplash-associated disorders with mild disability [5,18,38]. However, our results contrast with some findings involving chronic cervical pain in which the cause was not controlled [7] or involved trauma [12,17,39-41]. In a group of 30 patients with chronic neck pain, Revel et al. [7] noted error scores almost double in magnitude (compared with an age-matched group of healthy individuals), indicative of significant impairments. Heikkilä and Åström [12] and Heikkilä and Wenngren [39] found significantly larger HRA errors in whiplash groups than in healthy controls. Overall, differences observed were not as great as those reported originally by Revel and colleagues [7]. Using a different measuring device, Loudon et al. [40] examined a small whiplash group with chronic symptoms, and reported that they had larger mean position-sense errors than did healthy individuals. In a study of patients with idiopathic or traumatic neck pain by Sjölander et al. [17], larger repositioning errors were found in patients with chronic neck pain than among asymptomatic subjects. These effects were more pronounced for patients with trauma than for those with insidious neck pain. The authors did not find any systematic over- or under-estimation among patients. They suggested that increased repositioning errors observed in chronic neck pain are a result of poor position sense due to disturbed proprioceptive input, rather than of systematic bias in motor control systems at central levels.

In contrast, and in concordance with our findings, Rix and Bagust [5] observed no significant differences in repositioning accuracy between groups with chronic, nontraumatic neck pain, when compared with control groups, except for mean global error scores following flexion. Also, Teng et al. [18], who investigated 20 patients with chronic neck pain, reported that history of chronic neck pain did not correlate with cervicocephalic kinesthetic sensibility in middle-aged adults. Edmondston et al. [42] investigated 21 subjects with postural neck pain, and 22 who were asymptomatic. They assessed subjects' ability to replicate self-selected 'good' posture. No significant differences in posture repositioning errors between groups were observed. The authors concluded that individuals with postural neck pain may have a different perception of "good" posture, but no significant difference in kinesthetic sensibility compared with matched asymptomatic subjects. Armstrong et al. [38] investigated 23 subjects with whiplash, and compared them with a matched control group. They found no differences in head and neck position sense between individuals with chronic whiplash-associated disorders and the controls. Woodhouse and Vasseljen [19] investigated 116 patients with traumatic or nontraumatic chronic neck pain. Cervical movements in the associated planes relative to the primary movement plane were reduced among the two groups with neck pain, in comparison with 57 asymptomatic controls. The authors postulated that changes were probably not related to a history of neck trauma, or to current pain, but more likely due to a history of long-lasting pain. They found no differences between groups in cervicocephalic kinesthetic sensibility.

In our study, we did find a statistically significant altered global HRA in the neck pain group for one of the test movements: flexion. However, due to the lack of homogeneity and variations in only one-sixth of the test movements, this might have limited clinical meaning and generalizability.

The relationship between head repositioning acuity and functional performance is clinically important. Investigators have observed larger repositioning errors in persons reporting greater problems with function (higher Neck Disability Index) [14,43] than in those with milder problems [14,38,43]. Larger repositioning errors in patients with chronic whiplash-associated disorders have also been observed, with dizziness and unsteadiness [14]. More recently, Owens et al. [44], using normal student volunteers, showed that a recent history of cervical extensor muscle contraction could produce HRA errors similar to those reported in patients with whiplash. The authors suggested that this supports the role of paraspinal muscles in sensorimotor dysfunction not necessarily related to trauma.

In patients with chronic neck pain, and under various testing conditions, investigators have observed considerable abnormalities in standing vertical posture [21-23,45,46]. There are, however, conflicting reports on characteristics of postural balance during quiet standing in these patients [24]. Others have pointed out large variations in postural performance among patients [21], or have recommended dynamic posturography on a sway-referenced force plate, for better quantification of postural problems [47]. In terms of postural stability and balance, considerable research is still needed to provide sound diagnostic tests appropriate for use in a routine, clinical setting.

### Clinical and Research Implications

Because functional and structural cervical pathology underlying chronic neck pain remain largely unclear, continued research is crucial. However, it has been suggested that deficits in proprioception and motor control, rather than chronic pain itself, might be prime factors limiting function and quality of life in affected patients [17,21].

Subgroups classified objectively, according to proprioceptive or nonproprioceptive etiology, could be the focus of further research. Moreover, future work also might consider whether methods used in our study could contribute to daily clinical care. We would like to see further investigations of measurements of functional proprioceptive impairment, and its association with pain. Future research should combine measures used in the present study with measures of disability (e.g., the Neck Disability Index). This is important, because kinesthetic deficits in the neck have been linked to severity of pain and disability. Furthermore, to support comparison of results among studies, we recommend standardization of hardware and protocols in studies using HRA, force platforms, and CSP. Lastly, we recommend investigation of effects of different treatment modalities on chronic neck pain, as measured by sensorimotor function tests, such as HRA and CSP.

## Conclusion

For patients with nontraumatic chronic pain, only one of six test movements showed global repositioning errors significantly larger than for controls. Likewise, postural measurements showed little impaired balance, and substantial variations were present within groups. These results contrast with some other studies of patients with either traumatic or nontraumatic neck pain. However, limiting factors in our own work mean that further investigation will be required to establish whether and how nontraumatic chronic neck pain influences proprioception in the neck.

## List of abbreviations

CSP: Computerized static posturography; HRA: Head repositioning accuracy; VAS: Visual analogue scale.

## Competing interests

The authors declare that they have no competing interests.

## Authors' contributions

PP, AH, ME, and DA participated in the design of the study and performed the analysis. AH, ME, and DA supervised data collection analysis. PP supervised the study process and wrote the manuscript. All authors revised and approved the final manuscript.
